# Recognizing Ethyl Chloride Neurotoxicity: Inhalant Abuse Hidden in Plain Sight

**DOI:** 10.7759/cureus.37795

**Published:** 2023-04-18

**Authors:** Robert Young, Cody Carter, Serge Cardinali, Zeryab Khan, Katelyn Bennett, Amy Jarosz, Jeffery Sobecki, Rupali Sharma, Ryan Martin

**Affiliations:** 1 Graduate Medical Education, OhioHealth Doctors Hospital, Columbus, USA; 2 Neurology, OhioHealth Doctors Hospital, Columbus, USA

**Keywords:** hydrocarbon toxicity, ataxia, inhalant abuse, neurotoxicity, ethyl chloride

## Abstract

Ethyl chloride is a common topical anesthetic. However, when abused as an inhalant, effects can range from headaches and dizziness to debilitating neurotoxicity requiring intubation. While previous case reports describe the short-term reversible neurotoxicity of ethyl chloride, ours show chronic morbidity and mortality outcome. During the initial evaluation, it is essential to consider the rising trend of commercially available inhalants being used as recreational drugs. We present a case of a middle-aged man presenting with subacute neurotoxicity due to repeated abuse of ethyl chloride.

## Introduction

Inhalants are volatile substances that have the potential for misuse due to their neuro-stimulatory and neuro-psychiatric effects. They have been utilized by young adults since the 1980s as “legally abused” drugs of abuse [1]. Ethyl chloride (EC; CH3CH2Cl) is commonly used in its aerosolized form to provide topical myofascial anesthesia when applied to the skin. When misused as an inhalant, ethyl chloride can cause signs and symptoms ranging from eye irritation and transient euphoria to hallucinations and loss of consciousness [1]. EC has rapid absorption into the blood from the lungs [1]. As a chlorinated hydrocarbon, it is lipophilic, allowing it to concentrate in the brain, producing neurologic effects. The pathophysiological effects of EC are not well understood, but studies have shown that acute solvent exposure leads to N-methyl-D-aspartate (NMDA) receptor inhibition [1]. We outline an unusual case of an abnormal constellation of neurologic symptoms not related to any specific neurodegenerative, motor, or autoimmune disease. Through rigorous clinical evaluation, we determined the cause to be EC inhalation. This case report provides insights into the diagnosis, pathophysiology, mortality, morbidity, and management of this common inhalant abuse.

## Case presentation

A 42-year-old male with a medical history of hypertension and type 2 diabetes mellitus presented with altered mentation, bilateral lower extremity weakness with paresthesia, dysarthria, and tremors of all extremities. His constellation of symptoms started approximately three weeks prior to presentation, and he was unclear in identifying an inciting event. He denied fever, chills, nausea, vomiting, headache, dysdiadochokinesia, and visual or auditory disturbances. Also, he denied any prior trauma-related event, such as a head injury. Family history was non-contributory. He admitted to recreational marijuana and tobacco use but denied alcohol consumption. The physical exam showed vital signs within normal limits and no focal sensory or motor deficits; however, a shuffling gait was noted, and he was unable to ambulate without support. CT scan and MRI without contrast of the brain showed no obvious acute infarct, swelling, or enhancement. MRI of the cervical, thoracic, and lumbar vertebrae showed chronic foraminal stenosis and vertebral degeneration. Complete blood counts, metabolic panel, vitamin B1, B9, B12, and serologic studies, which included microbial and autoimmune screening, were normal. Neurology was consulted for further workup of ataxia and acute encephalopathy. The electroencephalogram (EEG) showed occasional low amplitude irregular delta slowing during drowsy periods without epileptiform activity. A lumbar puncture was performed and showed that cerebrospinal fluid (CSF) cell count, 14-3-3 protein, and autoimmune screening were all unremarkable. On the fifth day of admission, the patient endorsed transient visual hallucinations, after which behavioral health was consulted. Upon further questioning, the patient admitted to recreational inhalation of EC up to three times weekly for the previous one and a half years for euphoria, with his last use reported to be one week before arrival. Over the next five days, his mentation improved. His hallucinations resolved, but he remained ataxic and required further physical therapy after discharge. Approximately two months after rehabilitation, during a primary care visit, the patient reported a mild improvement in his balance and that physical therapy was helping. He continued to have intermittent double vision and was referred for ophthalmology evaluation. For further assessment, ophthalmology initiated both serologic and imaging evaluation for the neuro-ophthalmologic disorder. Unfortunately, the patient passed two weeks following his visit with ophthalmology. The coroner’s report described the death cause as “hypertensive cardiovascular disease.” Extensive drug testing on the liver was negative. Chloroethane, a metabolite of ethyl chloride, was specifically tested for and was also negative.

## Discussion

EC appears to have a dose-dependent effect on the central nervous system (CNS). At low doses, it causes brief sensations of intoxication and euphoria. Higher doses can cause CNS depression, leading to ataxia, incoordination, impaired short-term memory, and unconsciousness [2-4]. Reported chronic neurologic effects include decreased reflexes, ataxia, tremors, speech difficulty, nystagmus, and hallucinations [2]. Additionally, there are cardiovascular effects, including ventricular and tachyarrhythmias. Due to its effect on the cardiovascular and neurologic systems, there have been reports of cardiac arrest [5,6]. Diagnosis of EC toxicity remains clinical, requiring a detailed history and physical examinations. An emphasis on social and psychiatric history as well as neurologic examination, will aid in the diagnosis. To date, there are no serologic or urinary tests to evaluate for EC abuse. Chronic inhalant abuse may have significant neuroimaging findings, including diffuse atrophy of the cerebrum, cerebellum, brain stem, sulcal widening, and ventricular dilatation [2-4,7-9]. Initial management includes removing the patient from exposure to the inhalant. Patients should have continuous cardiac monitoring and frequent neurologic assessments to monitor for cardiac arrhythmias and CNS depression. There are no reversal medications for EC toxicity. Most effects of EC toxicity are temporary, requiring supportive care. More severe cases may necessitate supplemental oxygen therapy and even mechanical ventilation, depending on the level of CNS depression. Resolution of symptoms commonly occurs within one week from exposure to EC but may take several weeks for more severe cases [8,9]. Patients are typically adolescents and should be screened for concomitant alcohol use and other drugs of abuse in addition to behavioral disorders. Long-term follow-up should include wellness visits to ensure continued cessation of EC abuse and treatment of coexisting psychiatric illness. All hydrocarbon toxicities can potentially cause ataxia, cognitive impairment, and arrhythmias to varying degrees based on their individual chemical properties [10,11]. A summary of more common inhalants of abuse can be found in Table 1.

**Table 1:** Commonly abused inhalants. Source: References [10-14]

Inhalant	Chemical	Symptoms of intoxication
Glues	Toluene, various hydrocarbons and ketones	Seizures, Headache, nausea, cognitive impairment, arrhythmia
Paint thinner	Toluene and various hydrocarbons	Nausea, renal failure, seizures, ataxia, arrhythmia
Spray paint	Toluene and alkanes	Slurred speech, ataxia, cognitive impairment, arrhythmia
Keyboard duster	Difluoroethane	Nausea, cognitive impairment, headache, arrhythmia
Gasoline	Various hydrocarbons	Nausea, vision loss, headache, seizures, arrhythmia
Lighter fluid	Butane	Cognitive impairment and arrhythmia
Permanent markers	Toluene and alkanes	Nausea, slurred speech, cognitive impairment, and arrhythmia
Correction fluid	Toluene and Trichloroethylene	Nausea, headache, ataxia, cognitive impairment, and arrhythmia
Nail polish remover	Acetone	Cognitive impairment and hyperglycemia
Carburetor cleaner	Acetone, Toluene, methanol, and Propane	Nausea, ataxia, vision loss, cognitive impairment, and arrhythmia
Room deodorizers (“Poppers”)	Amyl nitrite and other nitrites	Flushing, headache, nausea, methemoglobinemia
Whipped cream dispenser (“Whippets”)	Nitrous oxide	Polyneuropathy, Ataxia, and cognitive impairment
Degreaser	Trichloroethylene	Headache, ataxia, cognitive impairment

Given the broad differential of hydrocarbon-containing products, a thorough history and physical exam with an emphasis on any oral or nasal skin findings and patient smell may help narrow the differential diagnosis. Patients that abuse nitrite-containing compounds may present with methemoglobinemia or polyneuropathy due to pathways not affected by hydrocarbons [12]. Vapors containing methanol can lead to vision loss [13]. Volatile gas panels can help to rule out more commonly abused inhalants and may have local variations in what is tested, so it is essential to know what is included.

## Conclusions

The addictive potential of EC is unclear. However, with our patient’s history of polysubstance abuse, concerns for recidivism and medical noncompliance were present. His death illustrates that a structured and concerted effort from both psychotherapy and possible medical management was needed. Further investigation into pharmacotherapy due to the high morbidity and mortality associated with EC is needed.
